# Supplementary material for: Expression of Estrogen Receptor Alpha and Evaluation of Histological Degeneration Scores in Fibroblasts of Hypertrophied Ligamentum Flavum: A Qualitative Study
Source: Biomolecules. 2021 Nov 24;11(12):1752. doi: 10.3390/biom11121752 (PMC8698276; doi:10.3390/biom11121752)
Supplement: Supplementary file 1 [file biomolecules-11-01752-s001.zip › biomolecules-1430082-supplementary.pdf]

Table S1. Influence of additional orthopedic diagnoses on the histopathology (Okuda scores) among LSS patients.

Osteochondrosis	No (N=15)	Yes (N=23)	P.Overall
**Okuda degeneration of elastic fibers**			0.102
mild	6 (42.9%)	16 (69.6%)	
moderate	6 (42.9%)	7 (30.4%)	
severe	2 (14.3%)	0 (0.00%)	
**Okuda proliferation of collagen fibers**			0.262
mild	4 (28.6%)	8 (34.8%)	
moderate	8 (57.1%)	15 (65.2%)	
severe	2 (14.3%)	0 (0.00%)	
**Okuda loss of elastic fibers**			0.141
mild	5 (35.7%)	13 (56.5%)	
moderate	7 (50.0%)	10 (43.5%)	
severe	2 (14.3%)	0 (0.00%)	
**Structural instability**	**No (N=28)**	**Yes (N=10)**	**P.Overall**
**Okuda degeneration of elastic fibers**			0.584
mild	17 (63.0%)	5 (50.0%)	
moderate	9 (33.3%)	4 (40.0%)	
severe	1 (3.70%)	1 (10.0%)	
**Okuda proliferation of collagen fibers**			0.479
mild	10 (37.0%)	2 (20.0%)	
moderate	16 (59.3%)	7 (70.0%)	
severe	1 (3.70%)	1 (10.0%)	
**Okuda loss of elastic fibers**			0.252
mild	15 (55.6%)	3 (30.0%)	
moderate	11 (40.7%)	6 (60.0%)	
severe	1 (3.70%)	1 (10.0%)	
**Scoliosis**	**No (N=33)**	**Yes (N=5)**	**P.Overall**
**Okuda degeneration of elastic fibers**			0.697
mild	20 (60.6%)	2 (50.0%)	
moderate	11 (33.3%)	2 (50.0%)	
severe	2 (6.06%)	0 (0.00%)	
**Okuda proliferation of collagen fibers**			0.425
mild	12 (36.4%)	0 (0.00%)	
moderate	19 (57.6%)	4 (100%)	
severe	2 (6.06%)	0 (0.00%)	
**Okuda loss of elastic fibers**			0.475
mild	17 (51.5%)	1 (25.0%)	
moderate	14 (42.4%)	3 (75.0%)	
severe	2 (6.06%)	0 (0.00%)	

Continuous variables are described with count and percentages in parentheses. P values between absence and presence of the respective additional orthopedic diagnosis were calculated with Fisher's exact-test. One case of LSS patients could not be evaluated due to lack of material.

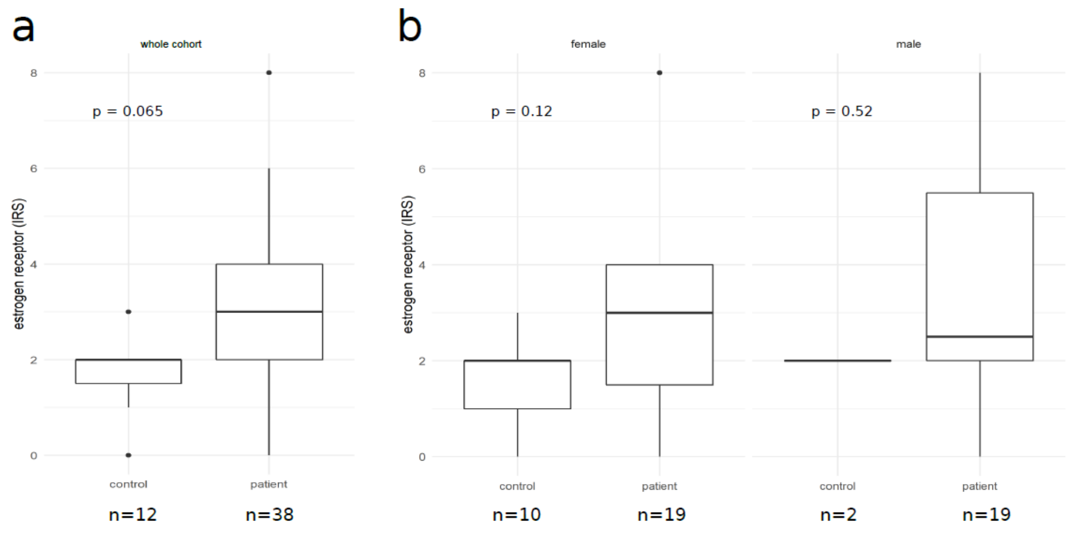

Figure S1. Box plots display ER α expression as IRS with respect to control or patient status in the whole cohort (**a**) and differentiated by sex (**b**). The boxes signify the first to third quartiles with the median as bar. The vertical lines indicate all values with the ends representing minimum and maximum values, outliers are depicted as dots. *p* values are calculated with the Kruskal-Wallis-test.
